# Curriculum and training needs of mid-level health workers in Africa: a situational review from Kenya, Nigeria, South Africa and Uganda

**DOI:** 10.1186/s12913-018-3362-9

**Published:** 2018-07-16

**Authors:** Ian Couper, Sunanda Ray, Duane Blaauw, Gideon Ng’wena, Lucy Muchiri, Eren Oyungu, Akinyinka Omigbodun, Imran Morhason-Bello, Charles Ibingira, James Tumwine, Daphney Conco, Sharon Fonn

**Affiliations:** 1 Ukwanda Centre for Rural Health, Stellenbosch University, PO Box 241, Cape Town, 8000 South Africa; 2 Centre for Rural Health, University of the Witwatersrand, Johannesburg, South Africa; 3 Department of Community Medicine, College of Health Sciences, University of Zimbabwe, PO Box A178, Avondale, Harare, Zimbabwe; 4 School of Public Health, University of the Witwatersrand, Johannesburg, South Africa; 5 Centre for Health Policy, School of Public Health, University of the Witwatersrand, Private Bag 3, Johannesburg, 2050 South Africa; 6 Maseno University School of Medicine, PO Box 333, Maseno, Kenya; 7 Department of Human Pathology, School of Medicine, College of Health Sciences, University of Nairobi, P.O. Box 19676, Nairobi, 00202 Kenya; 8 School of Medicine, Moi University, PO Box 4606, Eldoret, 030100 Kenya; 9 College of Medicine, University of Ibadan, Ibadan, Nigeria; 10 Department of Obstetrics and Gynaecology, University College Hospital, Ibadan, Nigeria; 11 Department of Obstetrics and Gynaecology, Faculty of Clinical Sciences, College of Medicine, University of Ibadan, Ibadan, Nigeria; 12 College of Health Sciences, Makerere University, PO Box 7072, Kampala, Uganda; 13 Department of Paediatrics and Child Health, School of Medicine, College of Health Sciences, Makerere University, P.O. Box 7072, Kampala, Uganda; 14 School of Public Health, University of the Witwatersrand, Wits Education Campus, 27 Saint Andrews Road, Parktown, Johannesburg, 2193 South Africa

**Keywords:** Healthcare providers, Healthcare workers, Mid-level workers, Primary healthcare, Educational models, Quality of healthcare, Curricula, Africa

## Abstract

**Background:**

Africa’s health systems rely on services provided by mid-level health workers (MLWs). Investment in their training is worthwhile since they are more likely to be retained in underserved areas, require shorter training courses and are less dependent on technology and investigations in their clinical practice than physicians. Their training programs and curricula need up-dating to be relevant to their practice and to reflect advances in health professional education.

This study was conducted to review the training and curricula of MLWs in Kenya, Nigeria, South Africa and Uganda, to ascertain areas for improvement.

**Methods:**

Key informants from professional associations, regulatory bodies, training institutions, labour organisations and government ministries were interviewed in each country. Policy documents and training curricula were reviewed for relevant content. Feedback was provided through stakeholder and participant meetings and comments recorded. 421 District managers and 975 MLWs from urban and rural government district health facilities completed self-administered questionnaires regarding MLW training and performance.

**Results:**

Qualitative data indicated commonalities in scope of practice and in training programs across the four countries, with a focus on basic diagnosis and medical treatment. Older programs tended to be more didactic in their training approach and were often lacking in resources. Significant concerns regarding skills gaps and quality of training were raised. Nevertheless, quantitative data showed that most MLWs felt their basic training was adequate for the work they do. MLWs and district managers indicated that training methods needed updating with additional skills offered. MLWs wanted their training to include more problem-solving approaches and practical procedures that could be life-saving.

**Conclusions:**

MLWs are essential frontline workers in health services, not just a stop-gap. In Kenya, Nigeria and Uganda, their important role is appreciated by health service managers. At the same time, significant deficiencies in training program content and educational methodologies exist in these countries, whereas programs in South Africa appear to have benefited from their more recent origin. Improvements to training and curricula, based on international educational developments as well as the local burden of disease, will enable them to function with greater effectiveness and contribute to better quality care and outcomes.

**Electronic supplementary material:**

The online version of this article (10.1186/s12913-018-3362-9) contains supplementary material, which is available to authorized users.

## Background

Achieving universal health coverage requires a well-trained and motivated health workforce, delivering a range of services. International experience suggests that mid-level health workers (MLWs) play an important role in addressing human resource shortages and improving health care access and equity, especially in low- and middle-income countries [1–5]. Africa’s health systems are dependent on services provided by MLWs, with initiation of new training programs in some countries and expansion of numbers of MLWs being trained in others in order to implement priority health programs. In 2007 this category of health care provider was identified in 25 out of 47 countries in sub-Saharan Africa [3]. Despite being the most cost-effective providers of primary and secondary health services, MLWs are often not included in health workforce planning and in some countries their roles are not formally regulated [5, 6]. They have been called doctor-substitutes, those who compensate for the scarcity of doctors especially in rural and underserved areas [3–5]. However, compared to doctors, they have higher retention in underserved areas, shorter training courses and lower dependence on expensive technology and investigations [4]. Whether MLWs are well-equipped to fulfil their responsibilities however, is uncertain. There have been several calls to review and update the curricula and training of MLWs, to ensure they gain the competencies necessary to make a significant impact in addressing twenty-first century healthcare needs [7–11].

Clinical MLWs include a range of cadres that carry out diagnostic and treatment functions conventionally thought of as the responsibility of doctors, usually in primary and secondary healthcare settings. They include clinical officers, health officers, medical assistants, *téchnicos de medicina* and *téchnicos de cirugia*, clinical associates and others who are trained to diagnose and manage common medical, maternal and child health (MCH) and surgical conditions [12]. Where nurses take on “medical” tasks such as making diagnoses, initiating treatment or performing anaesthesia, they may also be considered MLWs [5, 9]. Recently the term “associate clinician” has been adopted by MLWs^1^ as a unifying term in the professional development of this cadre [12].

Training programs for physician assistants, a profession with comparable concepts of clinical delegation and patient management, started in the USA in the mid-1960s, and have now been established in Europe, Australia and North America [13]. A recent systematic review of quality of care found that outcomes of numerous interventions in the areas of MCH, communicable and non-communicable diseases were similar whether carried out by MLWs (including midwives) or doctors, albeit that the level of evidence was low [6]. A Cochrane review similarly found that appropriately trained nurses could produce as high quality care as primary care doctors, with as good health outcomes for patients [14]. A more recent review found that nurse substitution for doctors in primary care has a positive effect on patient satisfaction, hospital admission and mortality [15]. The reviews emphasize the importance of good supervision and training relevant to purpose, based on an understanding that there is a chain that links effective learning to high-quality services and thus to improved health [16]. It is therefore important to examine the training of MLWs in Africa, to assess whether it is fit-for-purpose and whether improvements can be made to training and thus to quality of care.

The training programs for African MLWs were mainly developed in the mid-twentieth century to address physician shortages during colonial and immediately post-colonial periods of African history, based on the medical model of education at that time. Kenya for example has trained clinical officers since 1928, though the original certificate course was replaced with a three-year diploma in 1967 [17]. Globally the literature is silent on the appropriateness and relevance of curricula, teaching methodologies and training personnel for MLWs, though there is some indication that curricula and teaching methods may not be tailored to the practice of these workers to meet needs of the communities they serve [5].

The Lancet Commission on the Education of Health Professionals for the twenty-first Century did not specifically discuss the training of MLWs. With regard to medical, nursing, and public health training it noted that professional education has not kept pace with current health challenges. This was attributed to “fragmented, outdated, and static curricula that produce ill-equipped graduates” characterised by a mismatch of competencies to health needs, poor teamwork, and a narrow focus on technical, individual and hospital-oriented care [16]. The same criticism is likely to apply to the training of MLWs, but there is a paucity of literature that critically appraises the relevance or appropriateness of MLW training, aside from descriptions of focused training for specific, narrowly defined roles.

The purpose of this paper is to describe the education and training of MLWs in four countries in Africa, to establish what constitutes the criteria for their training at present, and to compare this to contemporary knowledge of appropriate educational methods in relation to their expected contribution to health care. The review presented here was conducted in Kenya, Nigeria, South Africa and Uganda. These countries were chosen on the basis of an existing collaboration amongst researchers from these countries, and because they represent Western, Eastern and Southern Africa which has a range of experience of training MLWs.

Just as the roles and nomenclature for MLWs varies, their training has also not been standardised across Africa. Thus the health workers studied in this review differed by country. The training of the following health workers was examined:

- Clinical officers (COs) in Kenya and Uganda
- Community Health Officers (CHOs) and Community Health Extension Workers (CHEWs) in Nigeria
- Clinical associates (ClinAs) in South Africa

Key health indicators of each country is provided in Table 1 below to situate these MLWs in their respective contexts.

**Table 1:** Details of MLW training and scope of practice in four countries

Nigeria	Uganda	Kenya	South Africa
*Ratio of physicians per 1000 population (latest available data)* ^*a*^			
0.376 (2009)	0.12 (2005)	0.199 (2013)	0.767 (2015)
*Maternal mortality ratio,* i.e. *maternal deaths per 100,000 live births (2015)*^*b*^			
814	343	510	138
*Under-five mortality rate* i.e. *deaths of children under 5 years per 1000 live births (Median estimates, 2016)*^*c*^			
104.3	53.0	49.2	43.3
*Nomenclature and scope of practice*			
Community Health Officers (CHOs) and Community Health Extension Workers (CHEWs) • CHOs and CHEWs constitute 42% of all human resources in PHC compared to 8% nurses. • CHOs spend 70% of their time in clinic facilities providing essential health services while CHEWS spend up to 50% of their time in these facilities working under supervision of the CHOs. • 90% of all deliveries performed in PHC are conducted by CHEWS. • CHOs can become administrative heads of primary care facilities, or take on other administrative and management roles on the public service.	Clinical officers (COs) • Most COs work in rural settings in primary care, performing patient assessment, disease management, triage, minor surgical procedures, and referrals to tertiary centres. They participate in community outreach, health education, screening and care coordination. • COs are not trained to manage emergencies whether obstetric, surgical, paediatric or medical • COs can undergo specialty training to become Psychiatric COs, Ophthalmic Cos, etc.	Clinical officers (COs) • COs offer a wide range of preventive and curative medical and surgical services, functioning quite independently at a range of levels in the health service though focussing particularly on clinics, health centres and district hospitals. Since the late 1970s COs could specialise via 2-year Higher Diploma courses in paediatrics, ophthalmology and other specialties, which has been extended to include ENT, anaesthetics, respiratory health, dermatology and reproductive health	Clinical Associates (ClinAs) • A new cadre, training of whom started in 2008, working under the supervision of doctors mainly in district hospitals, with a focus on management of common and chronic conditions, emergency care, skilled procedures, and inpatient care.
*Pre-entry requirements*			
• Both CHEWs and CHOs gained entry to study through five credit level passes in the Senior Secondary School Certificate examinations (or equivalent) taken after 12 years in the school system	▪ The minimum entry requirement was the Uganda Advanced Certificate of Education, taken after 12 years of schooling ▪ Qualified nurses could enter at the second year level of the Diploma	▪ The minimum entry requirement was a Kenya Certificate of Secondary Education, taken after completion of 12 years of schooling.	▪ The minimum entry requirement is a university entry exemption in the national senior certificate examination, taken after 12 years of schooling.
*Duration of pre-service training*			
▪ CHEWs: 3 years for National Diploma in Community Health ▪ CHOs were originally trained as nurses who then did post-basic training; a 4-year direct entry diploma was launched in 1990, which was subsequently upgraded to a Higher Diploma with plans to change this to a degree level course.	▪ 3 year Diploma in Clinical Medicine and Community Health ▪ Two year internship	▪ 3 year Diploma in Clinical Medicine and Surgery. ▪ One year of internship ▪ A 4 year BSc in Clinical Medicine was launched in 2010 at Mount Kenya University which expanded to 3 other universities.	▪ 3 year Bachelor of Clinical Medical Practice established in 2008 for training clinical associates
*Place of training*			
▪ Schools for CHOs were affiliated to universities while CHEWs were trained through Schools of Health Technology in each of the 36 states.	▪ 3 private institutions and 3 public schools that have trained large numbers of COs	▪ 27 accredited institutions that provide CO training including the Kenya Medical Training Centre, with its constituent colleges in various districts, five universities private and faith-based colleges	▪ University training through 3 medical schools, with ClinAs trained predominantly at district hospitals
*Educational content*			
▪ Diplomas were reviewed in 2006, with curricula adapted to include communications, ethics, health economics, information systems, human resources, and research methods	▪ Curriculum review in 1997 reoriented training towards preventive health and health promotion in addition to curative care. ▪ Contents include nutrition, health education, principles of PHC, maternal and child health, epidemiology, research methods, management, as well as anatomy, physiology, socio-psychology, dental health, internal medicine and pharmacology. ▪ Gaps in theoretical input on HIV and AIDS management, palliative care, new initiatives in malaria and TB. ▪ Limited practical exposure alongside theoretical teaching, especially in new approaches to common conditions	▪ CO curriculum reviewed in 2007, producing a common set of competencies and learning outcomes to be used across all CO training institutions, aiming to cover the range of medical problems encountered by COs. ▪ Respondents felt the training only prepared the COs for 50% of the conditions and issues dealt with in their actual workload ▪ Areas inadequately covered: basic sciences, research methodology, community health, HIV and AIDS, psychology, sociology and ethics. ▪ Bigger focus on health promotion and disease prevention, management of common conditions, not necessarily what COs were faced with in their workplaces	▪ Outcomes-based training according to a common curriculum framework with core competencies of clinical reasoning, investigative and therapeutic procedures appropriate for district hospitals, emergency care, clinical recordkeeping, ethics and professionalism, communication skills and counselling. ▪ The curriculum framework incorporates a set of common conditions presenting at district hospitals with a list of skills and procedures usually performed in district hospitals for which competency has to be achieved and demonstrated
*The trainers*			
▪ Range of health professionals (doctors, nurses, other MLWs) involved in teaching	▪ Training mainly by senior COs and medical technologists ▪ tutors were expected to teach with minimal resources due to paucity of teaching aids and reference materials	▪ Senior COs were responsible for training; ▪ Doctors involved in specialist training particularly for the Higher Diplomas	▪ Range of health professionals (doctors, nurses, other MLWs) involved in teaching

^a^Available from http://www.who.int/gho/health_workforce/physicians_density/en/

^b^Available from https://data.unicef.org/topic/maternal-health/maternal-mortality/

^c^Available from https://data.unicef.org/topic/child-survival/under-five-mortality/

This study is part of a broader research project concerned with the human resources for health crisis in Africa focusing on state services and non-profit services that act as proxy for the state (e.g. mission hospitals). Here we focus on the relevance and appropriateness of training of MLWs in equipping them for their public sector role in the selected African countries.

## Methods

This exploratory study, a two-phased, cross-sectional rapid appraisal, was carried out simultaneously in Kenya, Nigeria, South Africa and Uganda by locally-based research groups under supervision of the national members of the research collaboration. This paper combines the qualitative data and some of the quantitative survey results, with the aim to review the training and curricula of MLWs in Kenya, Nigeria, South Africa and Uganda, in order to ascertain where improvements can be made.

### Phase 1: Qualitative enquiry

A range of purposively-selected key informant interviews were conducted in each country. (See interview guide in Additional file 1). The interview questions and process were jointly developed during research planning and standardised across the countries. Participants were drawn from various professional regulatory bodies, professional associations, training institutions, labour organizations and relevant government ministries – Health, Finance, Labour and Education. In Nigeria key legislators also took part, the Chairpersons of the Health Committees of the Senate and the House of Representatives and a representative of a non-governmental organization (NGO) working on health in rural areas. Focus group discussions were also conducted with some of the participants. In addition, policy documents on education and curriculum for training of MLWs in each of the countries were assessed and examined for relevance and adequacy in content.

The information reported here from the interviews and document review focuses on those aspects of the research related to MLW training. For three of the countries, responses were based on many years of experience of these workers in their countries, whereas in South Africa they were based on the planned introduction of clinical associates, training of whom had recently commenced at the time of the interviews. Our inquiry focused on: entry requirements for training, training sites, curriculum content, educational methods and who the trainers were; generic questions about problems and suggested solutions including educational issues. All interviews were conducted in English, recorded and transcribed to improve accuracy and to support detailed analysis. Emergent and recurrent themes from the interviews were discussed and agreed upon by the investigators. In each country, there was triangulation across key informant interviews and focus group discussions, as well as between these and the document review. At the close of the study consultative stakeholder meetings were conducted in each country to validate and provide feedback on the research findings. The initial results of the surveys and interviews were presented, the proposed interventions discussed with themes and comments recorded for documentation.

### Phase 2: Quantitative enquiry

MLWs and district managers from urban and rural government district health facilities in Kenya, Nigeria and Uganda were requested to complete structured self-administered questionnaires. In South Africa, district managers were surveyed on their ideas about primary care nurses (who carry out similar functions as MLWs in other countries) since no clinical associates had yet graduated at the time of the study. Multistage stratified cluster sampling was used aiming for a sample size of 300 from each country. In Kenya we purposely selected one urban and one rural district from each province and then included all MLWs working in government health facilities in the selected districts. In Uganda 45 districts (out of a total of 77) were selected at random after stratifying by region, urban and rural districts, and better and worse functioning districts based on available health indicators and outcomes. All MLWs from the selected districts were included. In Nigeria we selected one state from each of the two health zones in both the North and South regions of the country. We selected every third Local Government Area (LGA) from the sampling frame in each of the selected states for a total of 240 LGAs. Every third person on the list of CHOs and CHEWs in the selected LGAs was included in the survey. The district health managers from each of the 52 districts in South Africa and the 72 districts in Kenya (using the district boundaries prior to 2002) were selected. In Uganda all district managers in the selected study districts were included. In Nigeria the primary health care coordinator, the LGA equivalent of the district manager, was included from each of the 240 selected LGAs.

The self-administered questionnaire was developed jointly by researchers from the four countries and piloted in each country. The final tool included 34 and 35 questions for district managers and MLWs respectively. Of relevance to this paper were questions on demographic characteristics; the adequacy of MLW training (yes or no); suggestions about how MLW training could be improved (three open-ended responses); and proposals to improve MLW performance (ranking the top 5 from a list of 15 identified in the literature). Data from the questionnaires was entered in Epi-Info (Epi Info™ 7. CDC, Atlanta, GA, USA, 2011) and analysed using Stata v11 (Stata Statistical Software: Release 11. StataCorp, College Station, TX, 2009). Open-ended responses were listed and then coded jointly by 4 researchers.

Ethical approval was obtained in each country by the lead institution in that country. (Complete information is provided at the end of the article.)

## Results

### Qualitative data

Table 1 displays the details of scope of practice, pre-entry requirements, duration of pre-service training, place of training and educational content, in the four countries. Their scope of practice was similar: MLWs provide general diagnoses and treatment in primary care clinics, health centres, and outpatient departments of district and mission hospitals. Those with specialist training may provide care in specific disciplines such as surgery, anaesthesia, psychiatry, HIV services and so on. There were commonalities across these countries: the MLW training programs recruited secondary school graduates; grade requirements tended to be lower than for medical degrees and more equivalent to entry for nursing education; the basic pre-service training took three years, with the option of further specialist training in Kenya and Uganda, or upgrading to a Higher Diploma in Nigeria. Training was mainly conducted through specific public sector training institutions established by Ministries of Health for MLWs.

MLWs were trained in basic diagnosis and medical treatment. In Uganda, a curriculum review in 1997 reoriented training towards preventive health and health promotion in addition to curative care. However, there were gaps identified in theoretical input on new developments such as management of HIV and AIDS, palliative care, and new initiatives in malaria and tuberculosis. In addition, there was limited practical exposure alongside theoretical teaching, especially in terms of new approaches to common conditions. In Nigeria, the diplomas were reviewed in 2006, with curricula adapted to include communications, ethics, health economics, information systems, human resources, and research methods. Kenya reviewed the CO curriculum in 2007, producing a common set of competencies and learning outcomes to be used across all CO training institutions, aiming to cover the range of medical problems encountered by COs. Despite this ambition, respondents felt the training only prepared the COs for 50% of the conditions and issues dealt with in their actual workload. Areas felt to be inadequately covered include basic sciences, research methodology, community health, HIV and AIDS, psychology, sociology and ethics. The older programs had a bigger focus on health promotion and disease prevention, and on management of common conditions, which was also not necessarily what COs were faced with in their workplaces. The three South African universities provided outcomes-based training according to a common curriculum framework with core competencies of clinical reasoning, investigative and therapeutic procedures appropriate for district hospitals, emergency care, clinical recordkeeping, ethics and professionalism, communication skills and counselling. The curriculum framework incorporates a set of common conditions presenting at district hospitals with a list of skills and procedures usually performed in district hospitals that they have to be competent in.

Training methods in the older programs relied on didactic teaching. More modern teaching and assessment methods such as adult and self-directed learning, problem-based learning, portfolios and Objective Structured Clinical Examinations (OSCEs) were uncommon. Teaching in the presence of patients on patient-based problems and practical demonstrations of history-taking, clinical examinations and interventions such as immunizations or wound-suturing were reported to be essential parts of these curricula. A recurring comment was that the educational content of training sessions were not aligned with the disease burden of the populations served and the kinds of conditions commonly presenting at rural health facilities. The training did not include methods of coping with day-to-day problems such as staff shortages, lack of equipment and medications. Respondents also indicated, particularly in Uganda, that tutors were expected to teach with minimal resources due to a paucity of teaching aids and reference materials. Since the clinical associates program in South Africa was newly developed it benefitted considerably from the experience of earlier programs in Africa. It was therefore able to draw on current educational thinking, incorporating intensive patient-based practical training, small group teaching, problem-based learning and self-directed learning.

Problems raised in the interviews related to training, as well as investments needed to improve quality of care are presented in Table 2. Key concerns were the quality and accreditation of training programs, numbers of students and trainers, and career progression. Participants requested more skills upgrading, continuing professional development (CPD), more support for specialisation and specialist MLW posts and other means of career progression. Participants wanted their training to include more skills in handling surgical and obstetric emergencies and practical procedures that could be life-saving and prevent disability particularly in obstetrics, trauma and surgery.

**Table 2:** Problems identified with MLW training and proposed solutions

Nigeria	Uganda	Kenya	South Africa
*Problems identified*			
• Inadequate numbers trained for volume of work	• Mainly manage common complaints, but not trained to manage emergencies – obstetric, surgical, some paediatric and medical • Lack of avenues for further training or CPD • MLWs dissatisfied with working conditions • Lack of ethical and professional behaviour: indiscipline and unwillingness to learn on part of students	• Gaps in training and specialisation • Trainers need upgrading in skills and methods of teaching • Minimal support for specialisation of MLWs or career progression	• Insufficient funding for trainers so fewer than required • Early days – the first cohort of 23 ClinAs qualified in 2010 followed by 93 in 2011, and are just getting established. • Hesitation on part of health science faculties to be involved
*Solutions proposed*			
▪ More emphasis on practical and curative aspects of work such as suturing wounds and surgical skills ▪ Improved training and supervision of primary care programs such as child health ▪ Upgrading training facilities ▪ Training of MLWs should take place at designated institutions and accreditation by National Board for Technical Education	▪ Standards suggested for improving teaching of MLWs such as better staff- student ratios; training on how to develop teaching plans and learning outcomes; how to motivate students; how to encourage professional behaviour. ▪ Review curriculum to impart more competencies and skills to MLWs	▪ Assessment by Clinical Officers Council before CO sent on internship ▪ Review curriculum ▪ Liaise with universities, Directorate of Personnel Management, professional associations to create clear career path e.g. BSc in Clinical Medicine, and in specialities ▪ CPD programs through accredited providers, associations etc. ▪ Government policy should change to focus on quality not just numbers trained	• Increased funding for better staff-student ratio • Higher level political commitment in support of ClinA training

### Quantitative data

In Kenya, Nigeria and Uganda, 975 MLWs were surveyed. No questionnaires were administered to MLWs in South Africa as there were no qualified clinical associates at the time of the study. Four hundred and twenty one district managers were surveyed in Kenya, Nigeria, South Africa and Uganda (Table 3).

**Table 3:** Characteristics of respondents - MLWs and district managers

	MLWs						District Managers							
	Kenya		Nigeria		Uganda		Kenya		Nigeria		Uganda		S Africa	
	n	%	n	%	n	%	n	%	n	%	n	%	n	%
Number of respondents	402		179		394		141		222		27		31	
Female	150	37.3	151	84.4	76	19.3	42	29.8	61	27.6	2	7.4	17	54.8
Male	252	62.7	28	15.6	318	80.7	99	70.2	160	72.4	25	92.6	14	45.2
Age Mean ± SD	31.5 ± 8.2		41.4 ± 7.4		37.2 ± 8.8		39.8 ± 7.7		33.2 ± 7.0		46.5 ± 6.5		51.9 ± 6.5	
Nature of district														
Predominantly rural	108	27.1	21	11.9	–	–	43	30.5	88	40.7	–	–	16	51.6
Mixture of rural and urban	279	70.1	104	58.8	–	–	92	65.2	80	37.0	-	-	11	35.5
Predominantly urban	11	2.8	52	29.4	–	–	6	4.3	48	22.2	–	–	4	12.9
Sector of work														
Public sector	356	89.2	173	97.2	–	–								
Mission & NGO	43	10.7	5	2.8	–	–								
Type of facility														
Health post/dispensary	37	9.3	15	8.4	–	–								
Clinic & Health centre	86	24.1	162	91	–	–								
District centre	243	61.1	–	–	–	–								
Referral hospital	22	5.5	1	0.6	–	–								

Most practicing MLWs surveyed (73–85%) considered their basic training to be adequate for the work they do (Table 4). However, when asked how the training could be made more relevant, many respondents indicated that there is a need to change the training approach (21–54%), offer training in additional skills (16–65%) and improve training institutions (15–18%).

**Table 4:** MLW responses on their training

	Kenya		Nigeria		Uganda	
	n	%	n	%	n	%
Is your basic training adequate for the work you do now?						
Yes	271	73.4	146	84.9	315	79.9
No	98	26.6	26	15.1	79	20.1
Suggestions on how to make associate clinician training relevant to their work						
Change training approach	182	53.7%	95	49.7%	57	21.0%
To be trained in additional skills	53	15.6%	43	22.5%	175	64.6%
Improve training institutions	52	15.3%	35	18.3%	2	0.7%
Degree track	40	11.8%	9	4.7%	3	1.1%
Management skills	11	3.2%	–	–	20	7.4%
More public health approach	–	–	9	4.7%	3	1.1%
Other	1	0.3%	–	–	11	4.1%
Total	339	100.0%	191	100.0%	271	100.0%

District managers were far less positive about the adequacy of training of MLWs; a minority (37–47%) felt the training is adequate for the work that these cadres do. (Table 5) Suggestions by district managers for how the training could be made more relevant were to change the training approach (39–63%), offer training in additional skills (15–39%) and improve training institutions (5–23%).

**Table 5:** District managers’ responses on MLW training

	Kenya		Nigeria		South Africa		Uganda	
	n	%	n	%	n	%	n	%
Is the training that MLWs receive adequate for the work that they do?								
Yes	64	47.4	87	43.7	9	37.5	10	37.0
No	71	52.6	112	56.3	15	62.5	17	63.0
District managers suggestions on how the training of MLWs could be improved								
Change training approach	75	45.2	286	62.6	14	31.1	18	39.1
To be trained in additional skills	25	15.1	3	0.7	17	37.8	18	39.1
Degree track	23	13.9	23	5.0	–	–	–	–
Management skills	21	12.7	–	–	1	2.2	8	17.4
More public or community health approach	9	5.4	–	–	5	11.1	–	–
Improve training institutions	9	5.4	105	23.0	–	–	1	2.2
Incentive/remuneration	–	–	18	3.9	–	–	–	–
Better selection	–	–	1	0.2	–	–	–	–
Make training appropriate to national priorities	–	–	–	–	7	15.6	–	–
Other	4	2.4	21	4.6	1	2.2	1	2.2

Respondents could give more than one response

## Discussion

MLW training programs in Africa offer an important avenue for scaling up human resources to meet health needs of communities as part of achieving universal health coverage, developing clinicians who are able to provide diagnostic and therapeutic services with lower entry qualification requirements and shorter training periods than for physicians. Expansion of these programs will require greater resources and more trainers, but must also address issues of quality and relevance. Increasing the contributions MLWs make to health care will require significant new investment to be made in their training, including trainers and facilities [4]. WHO has called for transformation and scaling up of health professionals’ education (HPE) through greater alignment between educational institutions and health systems, adapting curricula to evolving healthcare needs, accreditation of HPE programs and innovative expansion of faculty including community-based clinicians as educators [18]. MLWs must be part of that.

Common themes from our data resonate with the WHO recommendations [18]: the need to modernise curricula and incorporate innovative approaches to learning and teaching, to align the content of education programs to the burden of disease faced by MLWs in the workplace and to ensure appropriate accreditation of these programs. Feedback from MLWs and district managers in Kenya, Nigeria and Uganda suggests that there are significant deficiencies in training content and educational methodologies. The MLWs surveyed wanted changes in their training methods, updating of the skills of their trainers and supervisors, upgrading of their training facilities and improvement in hands-on clinical practice during training. Training curricula for doctors have been criticised for not being reflective of emerging population health needs with “insufficient alignment between the priorities and planning of the health and education sectors, (and) imbalanced distribution that disadvantages rural and poor urban populations” [19]. Calls for a transformative approach to medical education, one that is defined by a commitment to social responsibility, inter-sectoral engagement, relevance to disease profiles and emerging public health problems [19], apply equally to training of MLWs. A fundamental shift in educational strategy is essential if health professionals are to acquire the necessary skills in mobilising knowledge and deploying critical thinking to patient care and population health [16].

### Alignment of training institutions with health systems

The greater alignment between educational institutions and health systems should extend to MLW training which mainly takes place outside of medical schools in training institutions created by Ministries of Health and Education for this purpose. South Africa is the exception. Participants in our study called for curriculum review and formal accreditation of MLW training, which should be standard processes in all HPE institutions [18], and which could be addressed through more formal incorporation of such training into university or other higher education institutional structures. Medical schools across Africa are adopting innovative, problem-solving, student- centred and community-based approaches to medical education [20, 21], which are equally appropriate for MLW pre-service and in-service training. Developing a regional or continental network of training programs with cross-country comparisons and peer review could provide additional support for this. The move in some countries from diploma qualifications to university degrees for MLWs will help them to link in much more with developments in medical schools, and thus to innovative approaches for HPE overall. This upgrading should be informed by deficits faced by MLWs trained at diploma level so that graduates are able to offer better care. More recently established MLW training programs have opportunities to innovate in the design of their teaching methods and curricula, while older programs can reflect on the achievements of the past, and to reorient their approaches towards achieving their stated goals and objectives.

Compared with medical training, MLW training programs accept individuals with lower levels of schooling, shorter training periods, with less reliance on hospitals and advanced technology [3, 8]. The inclination to lengthen training to address some of the gaps should be resisted since the content and teaching methods are more critical than the length of course. Huicho et al. [22] showed no differences in quality of child health care between health workers with shorter or longer duration of training, although they did not examine the nature of the training and focused on very specific protocol-based tasks. Changing to competency-based education would allow for variable lengths of training, accommodating the skills and abilities of individual learners, and ensuring that trainees are assessed for competency before becoming independent practitioners [23].

### Curriculum review

Participants were concerned about the limited focus of their training curricula. Reviews of the content of curricula and training programs have not occurred recently in line with current thinking on health professions’ education, and in-depth analysis of these are clearly needed. The gaps in clinical skills, particularly related to the major causes of disease burden in Africa (maternal and child mortality, infectious diseases, trauma and violence) and newer challenges (HIV/AIDS and emerging chronic diseases), are significant and need to be addressed. Where reviews and changes in curricula have taken place, these have often led to the addition of theoretical content such as in health economics, ethics and research methods, rather than inclusion of problem-solving and case-study methods that prepare MLWs to deal with problems faced at health facilities. The solutions proposed by participants in this study include making the training more fit-for-purpose, with better regulation of training and monitoring progression against established standards. Participants requested that the content of programs be grounded in the disease burden of the populations served and should reflect the range and complexity of conditions they have to deal with at district level facilities. Country-specific morbidity profiles and health care needs should be the basis for addressing deficiencies in knowledge and skills. Teaching of research methods, economics and ethics could flow from the problems arising at facility level, rather than from theory. Benefits for whole health care teams would come from implementing care delivery models that best serve the local population health needs, using interventions known to be cost-effective, and that are taught by those with the appropriate skills and experience of those needs and models [6].

### Scope of practice

Limiting the scope of what MLWs are trained to carry out, making them focus on preventive health and minor conditions, while at the same time not providing them with the skills to respond to emergencies, has implications for how useful they can be and to their own sense of efficacy, especially where referral systems are limited. Dovlo points out that limiting MLW training to minor procedures may lead to them becoming a “transit referral point”, and thus a potential bottleneck in emergency care [4]. General surgery at district hospitals is highly cost-effective relative to other interventions in sub-Saharan Africa and in comparison to referral hospitals because of the relatively low input costs related to infrastructure and the high level of the avertable disease and disability burden [24, 25]. Where MLWs work under supervision of district doctors, giving them surgical skills to manage emergency situations and surgical procedures, provides good value for money [26]. Requests for more specialist training and career progression were raised in our study, though some MLWs did have opportunities for further training in a number of disciplines such as otorhinolaryngology, ophthalmology and anaesthetics. In the South African model, competency in emergency medicine and trauma procedures are required for ClinAs.

### Sustaining performance and CPD

Managers’ perceptions that MLW performance could improve and be sustained have highlighted the importance of follow-up training and CPD [4], concerns also expressed by MLW participants in this study. Adult learning methods that are life-long, experiential, reflective and linked to career progression, are more effective than didactic teaching when used for CPD. There is now stronger evidence emerging of the importance of training in the location of future work. Rural placements that are well-structured and supervised are better at equipping health professionals to work in the same environments [27]. Specialist outreach also facilitates supervision and mentorship of MLWs training in rural areas [7]. Supportive supervision focused on clinical mentorship rather than mainly on administration at both pre-service and in-service training is critical, yet a review of primary care supervision in developing countries found that clinical supervision (checking diagnostic and/or therapeutic skills) was uncommon [28]. Educators also need updating through CPD since many are out-of-touch with learning techniques and skills required for clinical curative primary care [5]. Many Health Professional Councils or Boards in African countries now have a requirement to demonstrate annual CPD credits for continued registration, which should apply to MLWs as well.

### Investments required

Rather than being treated as a stop-gap in primary and secondary health services, MLWs should be recognised for the essential frontline health workers they are. Their training and curricula require improvement to enable them to carry out the functions that they do with greater effectiveness and with regard to better quality care and enhanced outcomes. National governments should take a lead to ensure there is an enabling regulatory and accreditation framework for training, and to resource, guide and support educational institutions to upgrade training (quantity, quality and relevance), at both pre-service and in-service levels [16]. Targeted investments in infrastructure, faculty and training are necessary, and early collaboration with appropriate, socially accountable medical and nursing faculties could provide the necessary support for new programs.

Development and provision of appropriate trainers is critical in this. The slow growth of the health workforce lags behind population growth and increased health need in Africa [29], with the shortage of senior clinical educators – doctors and nurses – undermining training of all health staff as well as provision of services. Recent developments in many African countries of placing Family Physicians and Family Medicine training at district hospitals could facilitate incorporation of MLWs as valued members of district health teams, allowing them to develop practical skills under supervision, at the same time freeing up doctors for other more complex work [29, 30].

The costs of training MLWs and supporting them in practice should be compared with equivalent costs for physicians, to ensure that interventions are cost-effective, at the same time as being relevant and enhancing quality of care. Such an approach is essential given the significant economic, political, sociocultural and other external forces influencing decision-making in the countries studied, as exemplars of Africa, while recognising that investment in education and job creation in the health sector will contribute to promoting economic growth [31].

The progressive upgrade of MLW training in Africa has led to increasing professionalization and subsequent establishment of degree programs and an international professional association. The creation of the African Network of Associate Clinicians (ANAC) is an important step forward in recognising MLWs as health professionals who are making a significant contribution to primary and secondary health care, rather than being just a stop-gap measure. ANAC could collaborate with the International Academy of Physician Associate Educators (IAPAE)^2^ and through this synergy promote the global recognition of this cadre of health professionals.

### Limitations

Our study provides useful insights on the appropriateness and relevance of MLW training in Africa, a topic that has received insufficient attention in the literature to date. However, as a cross-sectional rapid appraisal, the study also had a number of limitations. We were not able to undertake a comprehensive and detailed evaluation of MLW training and training institutions but relied on policy documents, curricula, selected key informant interviews, and surveys with district managers and a sample of MLWs. The study was restricted to four countries, capturing a range of different MLW programs and experiences, but may not reflect all countries in the region. The quantitative survey with MLWs was limited in size, particularly in Nigeria, but with good participation rates indicating that the responses should be representative. Future studies, with more resources, will address these limitations.

## Conclusion

It is clear that MLWs address a major health need in Africa. It is also clear however that attention needs to be given to the content and pedagogic approach in training MLWs to ensure that fit-for-purpose graduates are better able to meet the demands of the workplace and the expectations of both health service managers and communities. Careful curricular review in relation to the burden of disease and most common problems addressed by MLWs in each context, drawing on international medical education guidelines, should be part of the necessary strategy to increase the numbers of MLWs being trained in Africa.

## Additional file

Additional file 1: Interview Schedule. Interview guide containing questions used to direct discussions with key informants. (DOCX 13 kb)

## Abbreviations

ANAC: African Network of Associate Clinicians
CHEW: Community health extension worker
CHO: Community Health Officer
ClinA: Clinical associate
CO: Clinical officer
CPD: Continuing professional development
ENT: Ear, nose and throat
HIV: Human Immunodeficiency Virus
HPE: Health professions education
LGA: Local government area
MCH: Maternal and child health
MLW: Mid-level health worker
NGO: Non-Governmental Organization
OSCE: Objective structured clinical examination
USA: United States of America

## Footnotes

1 The term MLWs is used in this paper rather than “non-physician clinicians” to avoid defining them as non-physicians and because it is the term most commonly used in the literature. We recognize however that the term preferred by many clinical MLWs is Associate Clinicians.

2 International Academy of Physician Associate Educators http://iapae.com/
