# Supplementary material for: Curriculum and training needs of mid-level health workers in Africa: a situational review from Kenya, Nigeria, South Africa and Uganda
Source: BMC Health Serv Res. 2018 Jul 16;18:553. doi: 10.1186/s12913-018-3362-9 (PMC6048766; doi:10.1186/s12913-018-3362-9)
Supplement: Supplementary file 1 — Interview Schedule. Interview guide containing questions used to direct discussions with key informants. (DOCX 13 kb) [file 12913_2018_3362_MOESM1_ESM.docx]

**CARTA-MED MID-LEVEL WORKER STUDY: INTERVIEW SCHEDULE FOR KEY INFORMANTS**

**Title of key informant (e.g. policy-maker, manager) and organisation: ………………………………………………… ……………………………………………………….………..……………………………………………………….**

**Country: ………………………..**

1. Please describe what cadres are called mid-level health workers (MLWs) in your country:
2. Describe the health facilities they mainly work in (probe on public and private sector, NGOs):
3. Describe the roles of MLWs in the health sector of your country (probe on scope of practice):
4. Please describe the training the MLWs receive (probe on institution of training, relationship to Ministry of Health, duration of training, practical versus lectures, curriculum content, style of teaching):
5. When was the curriculum for MLWs last reviewed to your knowledge?
6. Describe any problems you are aware of with the MLW training:
7. Describe what you suggest could solve these problems:
8. Describe constraints faced by MLWs in their work in the health sector:
9. What is the career progression for MLWs (probe on specialist training or entry into medical training):
